# Characterization of *CDKN2A*(p16) methylation and impact in colorectal cancer: systematic analysis using pyrosequencing

**DOI:** 10.1186/1479-5876-10-173

**Published:** 2012-08-27

**Authors:** Michel P Bihl, Anja Foerster, Alessandro Lugli, Inti Zlobec

**Affiliations:** 1 Institute of Pathology, University Hospital Basel, Basel, Switzerland; 2 Institute of Pathology, Bern, Bern, Switzerland

**Keywords:** Colorectal cancer, *CDKN2A*, p16, Methylation, Pyrosequencing

## Abstract

**Background:**

The aim of this study is to analyse *CDKN2A* methylation using pyrosequencing on a large cohort of colorectal cancers and corresponding non-neoplastic tissues. In a second step, the effect of methylation on clinical outcome is addressed.

**Methods:**

Primary colorectal cancers and matched non-neoplastic tissues from 432 patients underwent *CDKN2A* methylation analysis by pyrosequencing (PyroMarkQ96). Methylation was then related to clinical outcome, microsatellite instability (MSI), and *BRAF* and *KRAS* mutation. Different amplification conditions (35 to 50 PCR cycles) using a range of 0-100% methylated DNA were tested.

**Results:**

Background methylation was at most 10% with ≥35 PCR cycles. Correlation of observed and expected values was high, even at low methylation levels (0.02%, 0.6%, 2%). Accuracy of detection was optimal with 45 PCR cycles. Methylation in normal mucosa ranged from 0 to >90% in some cases. Based on the maximum value of 10% background, positivity was defined as a ≥20% difference in methylation between tumor and normal tissue, which occurred in 87 cases. *CDKN2A* methylation positivity was associated with MSI (p = 0.025), *BRAF* mutation (p < 0.0001), higher tumor grade (p < 0.0001), mucinous histology (p = 0.0209) but not with *KRAS* mutation. *CDKN2A* methylation had an independent adverse effect (p = 0.0058) on prognosis.

**Conclusion:**

The non-negligible *CDKN2A* methylation of normal colorectal mucosa may confound the assessment of tumor-specific hypermethylation, suggesting that corresponding non-neoplastic tissue should be used as a control. *CDKN2A* methylation is robustly detected by pyrosequencing, even at low levels, suggesting that this unfavorable prognostic biomarker warrants investigation in prospective studies.

## Background

The *CDKN2A* gene is located on chromosome 9p21 and encodes p16INK4a, a protein that functions to inhibit CDK4 and 6 within the cell cytoplasm. In colorectal epithelium, increased expression of p16INK4a may result from mutation of *BRAF* leading to oncogene-activated cell senescence [1,2]. Reversal of the senescence phenotype can be accomplished by hyper-methylation of the promoter region leading to the subsequent development of sessile serrated adenomas and possibly to carcinomas with high-level CpG Island Methylator Phenotype (CIMP) [3].

*CDKN2A* promoter hypermethylation has been described in 12-51% of colorectal cancers and is often included in the panel of markers used to assess the CIMP phenotype [4]. Age-related methylation has been documented and likely represents a confounding factor in the assessment of tumor-specific methylation and subsequently the correlation of hypermethylation with outcome [5]. Most studies to date evaluating *CDKN2A* and patient outcome have done so using methylation-specific PCR or MethyLight assays [4]. To date, pyrosequencing has only infrequently been used for methylation analysis of genes involved in colorectal cancer progression, including *CDKN2A*. Pyrosequencing is a valuable tool for methylation analysis for several reasons. It is a quantitative method that generates a percentage of methylation at a given CpG site by calculating the ratio of C/T (methylated to unmethylated C) in a sample [6]. Many different CpGs can be analysed at the same time, which allows specific sites and their potential clinical relevance to be studied separately. Internal controls can be included to determine inaccuracies resulting from incomplete bisulfite conversion. ln the case of *CDKN2A*, where a certain degree of age-related methylation in normal colonic mucosa may occur, the quantitative nature of the pyrosequencing method allows one to account for background methylation to provide a more accurate assessment of tumor-specific hypermethylation.

The aim of this study is to evaluate site-specific *CDKN2A* methylation patterns in both tumor and matched non-neoplastic tissues, amplification conditions and to propose a threshold value for *CDKN2A* methylation “positivity” by pyrosequencing. In a second step, we evaluated the impact of *CDKN2A* methylation on clinical outcome in a series of 432 patients with colorectal cancers.

## Methods

### Patients

Four hundred and thirty-two patients with primary colorectal cancer were entered into this study. Clinico-pathological information for each patient included age, tumor location, gender as well as pT, pN, tumor grade, histological subtype, vascular invasion, tumor growth pattern, tumor budding and the tumor border configuration. For all patients, information on survival could be obtained. Median follow-up and survival time were 55.0 months and 60.0 (95%CI46-74) months, respectively. Previously investigated molecular parameters included MSI status, *BRAF* and *KRAS* mutation, which were available for 395, 382, and 408 patients, respectively. Ethical consent was obtained from the local ethics committee.

### DNA isolation

Archival tissue blocks from each patient were obtained. DNA was extracted from both primary tumours and the adjacent non-neoplastic tissues from blocks with >70% tumor cell content using NucleoMag 96 Tissue Kit (Macherey Nagel) protocol and processed in the Xiril X-100 robot (Xiril, Hombrechtikon, Switzerland). Appropriate regions of interest were identified from H&E slides, marked using two different colors by an experienced gastrointestinal pathologist (A.L.) and macrodissected using a punching instrument.

### DNA denaturation and bisulfite conversion

DNA denaturation and bisulfite conversion were processed into one-step using the EZ-96 DNA Methylation-GoldTM Kit (Cat. No. D5007, ZYMO RESEARCH CORP.). Briefly, 130 μl of the CT conversion reagent were added to 20 μl of each DNA sample in a conversion plate. The plates were sealed with the provided film and the conversion plate transferred to a thermal cycler running under the following steps: 98°C for 10 minutes, 53°C for 4 hours, storage at 4°C. 400 μl of M-Binding Buffer were added to the wells of a Silicon-A™ Binding Plate. After transfer to the wells of the Silicon-A™ binding plate, samples were centrifuged. Each well of the plate was washed using 400 μl of M-wash buffer. After centrifugation, 200 μl of M-Desulphonation buffer was added to each and the plate was placed at room temperature for 20 minutes followed by centrifugation (3,000 x g for 5 minutes). Two consecutive steps including washing (400 μl of M-wash Buffer) and centrifugation (3,000 x g for 5 minutes) were performed. The Silicon-A™ binding plate was placed onto an elution plate and 30 μl of M-Elution buffer was directly added to each well. 5 minutes later, the samples were centrifuged at 3,000 x g for 3 minutes to elute the DNA. For control of DNA methylation, Universal Methylated Human DNA Standard was used (Cat. No. D5011, ZYMO RESEARCH CORP).

### Polymerase chain reaction (PCR)

The following primers for *CDKN2A* were used: Forward ATGGAGTTTTYGGTTGATTGGT (region: 21964802–21964781), reverse CCCRCCATCCCCTACTCC (region: 21964655–21964638); nested forward ATGGAGTTTTYGGTTGATTGGT, Reverse Biot-CCCTCTACCCACCTAAAT (region: 21964682–21964665), sequence primer GGAGTTTTAGGTTGATTGGTT. Amplicon length was 98 bp. For all 432 patients the methylation status of *CDKN2A* was the result of a first and semi-nested PCR. Each PCR began with a unique denaturation step for 11 min at 95°C followed by 45 cycles including denaturation, annealing and elongation at 95°C for 20 sec, 52°C for 15 sec, and 72°C for 20 sec respectively. The final elongation step was done at 72°C for 7 min.

### Preparation of samples for pyrosequencing

Three μl of Sepharose beads were mixed together with 40 μl of binding buffer and 22 μl of water and mixed in an Eppendorf tube. Sixty μl of this mix was added to 20 μl of PCR products in a 96 well plate and agitated at 1400 rpm for 5 minutes. The PyroMark Q96 Plate was filled with 0.3 μM of sequencing primer in 40 μl of annealing buffer. The washes were performed using the vacuum station according to the manufacturer’s instruction. For annealing the samples to sequencing primers, the temperature was increased to 80°C for 2 minutes and then left to cool at room temperature for 5 minutes. Plates were then ready for processing in the PyroMarkQ96 instrument.

### Pyrosequencing

**Figure 1:**
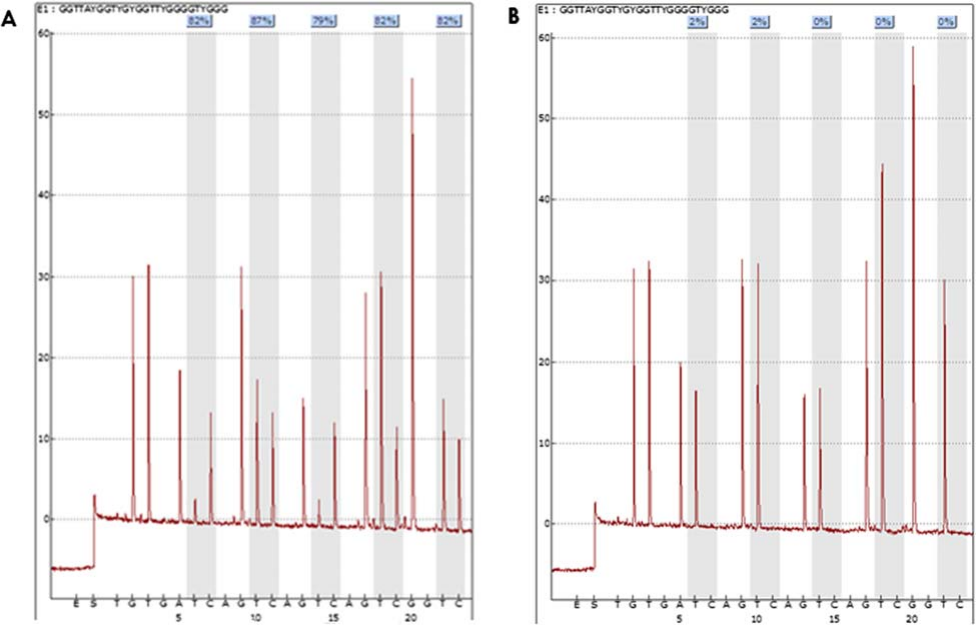
**Representative pyrograms with PyroMarkQ96 showing percentage of methylation at each of five CpG sites evaluated.A**) Highly methylated tumor sample with an average methylation percentage of (82% + 87% + 79% + 82% + 82%)/5 = 82.4%. **B**) Corresponding non-methylated non-tumoral tissue from the same patient as in A with the following methylation pattern: 2%, 2%, 0%, 0%, and 0%.

Pyrosequencing of the purified single stranded PCR products and CpG site quantification was accomplished by the PyroMarkQ96 and related software (Qiagen). The sequence to analyse was TTAYGGTYGYGGTTYGGGGTYGGGTAGAGGAGGTG and contained the densest region of CpG sites within our amplicon. The five CpG sites were investigated in both neoplastic and corresponding non-neoplastic tissues. Each CpG site was assigned a percentage of methylation by evaluating the C/T ratio. The average percentage of methylation across these 5 CpG sites was obtained. The tumor-specific methylation was calculated by subtracting the average% methylation of the normal mucosa from the average% methylation of tumor. Representative pyrograms are shown in Figure 1.

### Background and PCR cycle number

**Figure 2:**
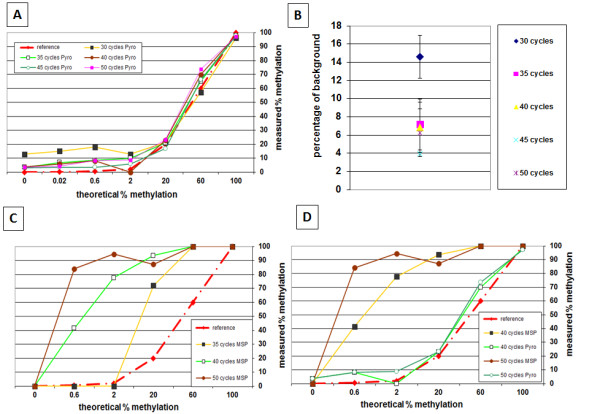
**A): Serial dilutions of 100% and 0% methylated DNA were combined and pyrosequencing used to analyse the measured and theoretical percentage of methylated DNA as a function of the number of PCR cycles.** A strong correlation between the measured and theoretical percentage of methylation was observed, particularly for PCR cycle numbers of 35 or more. **B**) Influence on methylation background as a function of PCR cycle numbers. **C**) In contrast to pyrosequencing, only a poor correlation between the measured and theoretical percentage of methylation was observed using methylation-specific PCR (MSP) which also showed a considerable dependency on PCR cycle number. **D**) Comparison of measured and theoretical percentage of methylation for 40 and 50 PCR cycles obtained using pyrosequencing and MSP.

Taking into consideration that *CDKN2A* methylation may occur in normal tissues (A-type methylation) as an age-dependent phenomenon, an appropriate cut-off score should be assigned above which “hypermethylation” can be defined [5]. To investigate this, the experimental background of the pyrosequencing method using samples prepared by mixing DNA obtained from peripheral blood leukocytes of healthy donors (unmethylated) and from commercially available 100% methylated DNA (Zymo Research D5011) was tested. Different concentrations of methylated p16 DNA were obtained by performing serial dilutions. The measured percentage of methylation was analysed by pyrosequencing using the PyroMarkQ24 instrument and compared to each theoretical percentage (ranging from 0-100%) (Figure 2A). The effect of different amplification conditions (30 to 50 PCR cycles) was also tested. A strong correlation between the measured and theoretical percentage methylation was observed. For 30 PCR cycles, the correlation was acceptable but a higher background methylation, up to 20%, was observed. With 35 cycles or more, background methylation levels between 0 to 10% were consistently observed (Figure 2B); 45 cycles showed the least degree of background.

### Comparison with methylation-specific PCR

The percentage of methylation for the same samples was also evaluated using methyl specific primer (MSP) amplification as established by Herman et al. [7]. Table 1 compares the genomic coordinates used in this study with those described by Herman et al. Interestingly the results obtained with this method were highly dependent on the number of PCR cycles performed (Figure 2C). The percentage of methylation given by this method was consistently higher than expected. For example, amplification with at least 40 cycles led to a methylation status of >80% although the percentage of the sample is 2%. A direct comparison between the methylation status obtained using pyrosequencing and MSP amplification for 40 and 50 amplification cycles was represented in Figure 2D.

**Table 1:** **Comparison of primers and genomic coordinates used in this study for methylation-specific PCR in comparison to Herman et al.**[7]

		**Position/AB060808.1**	**Position / Start codon**	**Sequence**
**CDS p16/CDKN2A exon 1**		**192221- > 192072**	**0 - > 149**	**ATGGAGCCGG…..GCCGATCCAG**
**This study**	**F1**	**192197- > 192176**	**24 - > 45**	**ATGGAGCCTTCGGCTGACTGGC**
	**R1_primer**	**192050- > 192033**	**171 - > 188**	**CCCGCCATCCCCTGCTCC**
	**R2_primer**	**192077- > 192060**	**144 - > 161**	**CCCTCTACCCACCTGGAT**
**Herman**	**F1**	**192301- > 192278**	**−80 - > −57**	**TCACCAGAGGGTGGGGCGGACCGC**
	**R1**	**192172- > 192151**	**49 - > 70**	**TTAACAAAAAAAAAAAACTAAACTCCTC**

### Statistical Analysis

Differences in the percentage of methylation between normal and tumor tissues at each CpG site were analysed using the non-parametric Wilcoxon’s Rank Sum test. Associations between methylation and categorical clinico-pathological features were investigated using the Chi-Square or Fisher’s Exact tests. For age and tumor size, differences between CDKN2A methylation positive and negative tumors were analysed using Wilcoxon’s Rank Sum Test. Kaplan-Meier plots and the log-rank test was used to analyse differences in survival time between patients with methylation positive and negative tumors un a univariate setting. Cox proportional hazards regression models were used in a multivariable setting to test the independent contribution of each variable to outcome after adjustment for the remaining potential confounding variables. Hazard ratios (HR) and 95% confidence intervals were used to determine the effect of each variable on outcome.

## Results

### CpG site-specific analysis of *CDKN2A*

**Figure 3:**
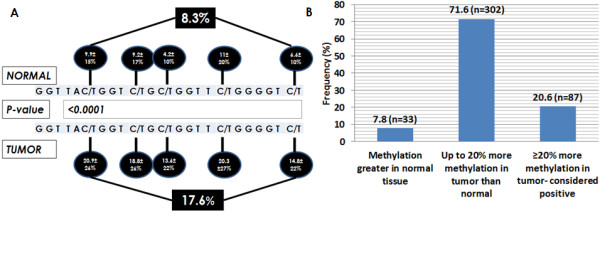
**A) Mean percent-methylation ± standard deviation of each CpG site within the region analysed.** The average methylation across all sites was 8.3% in normal mucosa and 17.6% in colorectal cancer. The percent-methylation at each CpG site is significantly (p < 0.0001, all) larger in the tumor compared with normal tissue. **B**) Distribution of methylation in normal tissues and tumor. 7.8% of all cases should a greater percentage of methylation in normal tissues than in tumor. Most cases showed slightly more methylation in tumor than normal while 20.6% of cases were considered methylation positive (at least 20% more methylation in tumor).

In a first step, colorectal cancers and corresponding normal tissue from 432 patients were evaluated for *CDKN2A* methylation. Five different CpG sites were assessed. In 10 cases, no evaluation of tumor could be made; the total number of patients successfully evaluated was thus 422. The mean ± SD percentage methylation at each CpG site was analysed in both normal mucosa and tumor. Results are shown in Figure 3A. In normal tissue, at each site percentage of methylation was 9.9%, 9.2%, 4.2%, 11% and 6.6%, respectively, while almost twice that in tumor, namely 20.9%, 18.8%, 13.6%, 20.3% and 14.8%. Tumors were significantly more methylated than normal tissues at each CpG site and on average across all sites (normal: 8.3% versus tumor: 17.6%) (p < 0.0001, all). No site was specifically correlated with patient age in normal tissue.

### Methylation differences between tumor and normal mucosa

The distribution of *CDKN2A* methylation differences between tumor and normal mucosa are shown in Figure 3B. Considering a background methylation rate of 10%, (see 2.7), 92.2% of all cases had equal or greater methylation in tumor compared to normal tissue, while 33 cases (7.8%) were more highly methylated in normal mucosa. Using a threshold value of 20% difference between tumor and normal tissue, 87 patients (20.6%) were considered methylation-positive.

### *CDKN2A* methylation and clinico-pathological and molecular data

Methylation of *CDKN2A* in colorectal cancers was significantly more frequent in right-sided colon cancers (p < 0.0001), as well as those with mucinous histology (p = 0.0209), higher tumor grade (p < 0.0001) and lymph node metastasis (p = 0.0335). Although methylation was not associated with *KRAS* mutation (p = 0.565), a strong relationship between *BRAF* mutation and *CDKN2A* methylation was observed (p < 0.0001). Specifically 31.2% of methylated cases showed *BRAF* mutation in comparison to only 5.9% of negative cases. In addition, methylation was found more frequently in MSI-H (23.5%) than MSS/MSI-L (13.4%) cancers (p = 0.0252) (Table 2).

**Table 2:** **Association of*CDKN2A*methylation and clinico-pathological features in colorectal cancers (n = 422)**

**Feature**		***CDKN2A*N(%)**		**P-value**
		**Negative (335; 79.4%)**	**Methylated (87; 20.6%)**	
Age	Mean (range)	69.3 (37–95)	68.8 (39–89)	0.7292
Tumor size	Mean (range)	49.3 (5–160)	53.5 (4–170)	0.1265
Tumor location	Left-sided	232 (69.5)	40 (46.5)	<0.0001
	Right-sided	102 (30.5)	46 (53.5)	
Gender	Female	181 (54.0)	48 (55.2)	0.849
	Male	154 (46.0)	39 (44.8)	
Histological subtype	Non-mucinous	19 (5.7)	11 (12.6)	0.0209
	Mucinous	316 (94.3)	76 (87.4)	
Tumor grade	G1-2	317 (96.7)	74 (85.1)	<0.0001
	G3	11 (3.4)	13 (14.9)	
pT classification	pT1-2	75 (22.9)	14 (16.1)	0.1711
	pT3-4	253 (77.1)	73 (83.9)	
pN classification	pN0	180 (55.9)	37 (43.0)	0.0335
	pN1-2	142 (44.1)	49 (57.0)	
Vascular invasion	Absent	238 (72.6)	54 (62.1)	0.0568
	Present	90 (27.4)	33 (37.9)	
Invasive margin	Pushing	101 (30.9)	21 (24.4)	0.242
	Infiltrating	226 (69.1)	65 (75.6)	
Peritumoral lymphocytic inflammation	Absent	251 (76.5)	66 (75.9)	0.897
	Present	77 (23.5)	21 (24.1)	
*KRAS* (codon 12/13)	Wild-type	227 (69.2)	58 (72.5)	0.565
	Mutation	101 (30.8)	22 (27.5)	
*BRAF* (codon V600E)	Wild-type	287 (94.1)	53 (68.8)	<0.0001
	Mutation	18 (5.9)	24 (31.2)	
MSI status	MSS/MSI-L	272 (86.6)	62 (76.5)	0.0252
	MSI-H	42 (13.4)	19 (23.5)	

### Effect of *CDKN2A* methylation on survival

**Figure 4:**
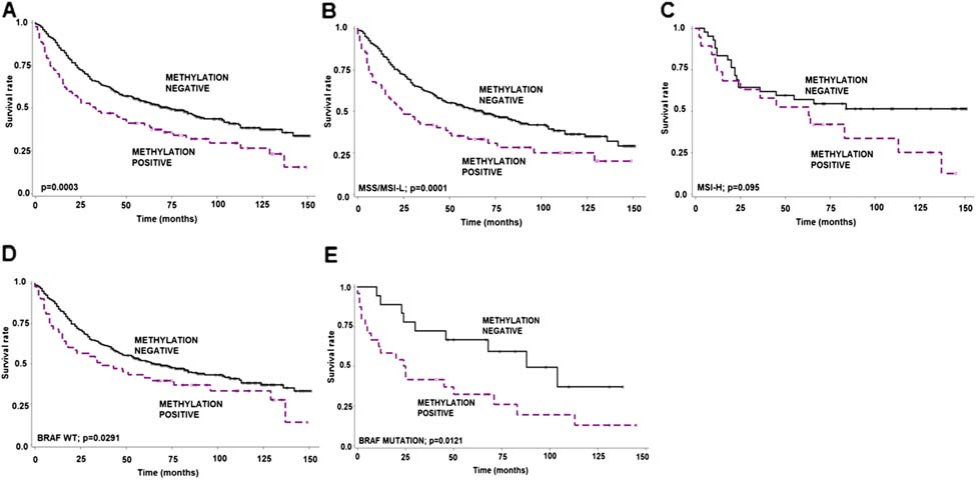
**Kaplan-Meier survival curves showing the unfavorable prognostic impact of *CDKN2A *methylation positivity in (A) all patients and in cases with (B) microsatellite stable (MSS/MSI-L), (C) microsatellite instability-high (MSI-H), (D) BRAF wild-type (WT) and (E) BRAF mutation.**

The prognostic effect of *CDKN2A* could be assessed in all 422 patients. Methylation led to a strong negative effect on survival time (p = 0.0003, log-rank; Figure 4A). This is additionally highlighted by a HR (95%CI) of 1.6 (1.2-2.1) indicating that patients with methylation-positive tumors have a 60% increased risk of death in comparison to methylation-negative patients. This association is maintained in patients with MSS/MSI-L cancers (p = 0.0001; Figure 4B) whereas the effect was non-significant in patients with MSI-H tumors (p = 0.095; Figure 4C). However, of the 19 patients with MSI-H/CDKN2A methylation positive cancers, 14 (74%) died of disease, in comparison to 20/42 (47.6%) of patients with MSI-H/CDKN2A methylation negative cancers.

Next we stratified the survival effect of CDKN2A methylation by BRAF status. In both BRAF WT (p = 0.0291) and BRAF mutated (p = 0.0121) tumors, CDKN2A methylation positivity had a significant and unfavourable effect on survival time (Figure 4 D, E).

We further performed multivariate analysis of CDKN2A using two different models. In the first, we analysed CDKN2A methylation status along with standard clinic-pathological data including age, pT, and pN classifications. In a second, we evaluated CDKN2A methylation along with MSS and BRAF status. In both models, methylation positivity maintained its significance unfavourable effect on outcome (Table 3).

**Table 3:** **Multivariate survival analysis of*CDKN2A*methylation in colorectal cancer**

	**MODEL 1**				**MODEL 2**		
		**HR (95%CI)**	**P-value**			**HR (95%CI)**	**P-value**
*CDKN2A*	Negative	1.0	0.0416	*CDKN2A*	Negative	1.0	0.0021
	Methylated	1.36 (1.1-1.8)			Methylated	1.66 (1.2-2.3)	
Age	Baseline	1.0		MSI	MSS/MSI-L	1.0	0.0273
	1-year	1.04 (1.02-1.05)	<0.0001		MSI-H	0.63 (0.4-0.9)	
pT	pT1-2	1.0	<0.0001	*BRAF*	Wild-type	1.0	0.2165
	pT3-4	1.98 (1.3-2.9)			Mutation	1.32 (0.9-2.0)	
pN	pN0	1.0	<0.0001				
	pN1-2	2.52 (1.9-3.3)					

### *CDKN2A* stratified by MSI status

Since methylation of *CDKN2A* occurs more frequently in MSI-H and shows the typical features characteristic of unstable cancers, we evaluated the relationship between *CDKN2A* methylation and prognostic factors in both MSS/MSI-L and MSI-H cases. Among patients with MSS/MSI-L tumors, methylation was still associated with right-sided disease (p = 0.0125), higher-tumor grade (p < 0.0001), lymph node positivity (p = 0.0207), and *BRAF* mutation (p < 0.0001. In contrast, in the MSI-H setting, methylation was linked to right-sided location (p = 0.013), higher pT classification (p = 0.0093), vascular invasion (p = 0.0326), the infiltrating growth pattern (p = 0.0281), *KRAS* mutation (p = 0.0356) and *BRAF* mutation (p = 0.0098) (Table 4).

**Table 4:** **Association of*CDKN2A*methylation and clinico-pathological features stratified by microsatellite instability (MSI) status**

**Feature**		**CDKN2A in MSS/MSI-L (n;%)**		**P-value**	**CDKN2A in MSI-H (n;%)**		**P-value**
		**Negative**	**Positive**		**Negative**	**Positive**	
		**(n = 272; 81.4%)**	**(n = 62; 18.6%)**		**(n = 42; 68.9%)**	**(n = 19; 31.2%)**	
Age	Mean (range)	69.5 (40–95)	69.9 (44–88)	0.788	68.4 (45–87)	68.2 (50–89)	0.9897
Tumor size	Mean (range)	48.0 (5–130)	49.8 (4–105)	0.523	54.9 (19–160)	62.8 (15–170)	0.3737
Tumor location	Left-sided	193 (71.2)	34 (54.8)	0.0125	24 (57.1)	4 (22.2)	0.013
	Right-sided	78 (28.8)	28 (45.2)		18 (42.9)	14 (77.8)	
Gender	Female	149 (54.8)	34 (54.8)	0.9932	20 (47.6)	10 (52.6)	0.7169
	Male	123 (45.2)	28 (45.2)		22 (52.4)	9 (47.4)	
Histological subtype	Non-mucinous	253 (93.0)	54 (87.1)	0.1142	39 (92.9)	16 (84.2)	0.2863
	Mucinous	16 (5.9)	8 (12.9)		3 (7.1)	2 (10.5)	
Tumor grade	G1-2	261 (98.1)	54 (87.1)	<0.0001	37 (88.1)	14 (73.7)	0.2608
	G3	5 (1.9)	8 (12.9)		5 (11.9)	5 (26.3)	
pT classification	pT1-2	59 (22.1)	13 (21.0)	0.8354	12 (28.6)	0 (0.0)	0.0093
	pT3-4	207 (77.8)	49 (79.0)		30 (71.4)	19 (100.0)	
pN classification	pN0	140 (53.4)	23 (37.1)	0.0207	26 (66.7)	10 (52.6)	0.3012
	pN1-2	122 (46.6)	39 (62.9)		13 (33.3)	9 (47.4)	
Vascular invasion	Absent	186 (69.9)	37 (59.7)	0.1193	35 (83.3)	11 (57.9)	0.0326
	Present	80 (30.1)	25 (40.3)		7 (16.7)	8 (42.1)	
Invasive margin	Pushing	69 (26.0)	12 (19.7)	0.2996	26 (61.9)	6 (31.6)	0.0281
	Infiltrating	196 (74.0)	49 (80.3)		16 (38.1)	13 (68.4)	
Peritumoral lymphocytic inflammation	Absent	211 (79.3)	51 (82.3)	0.6037	27 (64.3)	13 (68.4)	0.7529
	Present	55 (20.7)	11 (17.7)		15 (35.7)	6 (31.6)	
*KRAS* (codon 12/13)	Wild-type	185 (69.0)	41 (68.3)	0.9161	26 (65.0)	14 (93.3)	0.0356
	Mutation	83 (31.0)	19 (31.7)		14 (35.0)	1 (6.7)	
*BRAF* (codon V600E)	Wild-type	245 (96.8)	42 (75.0)	<0.0001	30 (79.0)	8 (44.4)	0.0098
	Mutation	8 (3.2)	14 (25.0)		8 (21.0)	10 (55.6)	

## Discussion

In this study we evaluated *CDKN2A* methylation using pyrosequencing on a large cohort of 422 patients with colorectal cancer. Using both primary tumors and matched non-neoplastic tissues, we analyzed individual CpG sites and different amplification conditions. Additionally, we highlight the negative and independent prognostic effect of *CDKN2A* methylation on prognosis in patients with colorectal cancer using pyrosequencing.

Our results show acceptable levels (<10%) of background *CDKN2A* methylation with ≥35 PCR cycles; lowest levels were reached at 45 cycles. The predicted and observed degrees of methylation found after serial dilutions with methylated and non-methylated DNA show a tight correlation at these amplification conditions, while even low levels could be accurately detected using 45 PCR cycles. A high number of amplification cycles is often necessary for pyrosequencing. Since both biotinylated template strands and unincorporated biotinylated primers can be captured on the streptavidin-coated beads, a high number of PCR cycles ensures that the biotinylated primer does not itself act as an additional sequencing primer thereby interfering with the subsequent sequencing reaction [6].

Although the average percentage of methylation in non-neoplastic tissues was <10% across all CpG sites, in some cases it could reach >90%. This non-negligible methylation pattern suggests that the normal corresponding mucosa must be used as a control in the assessment of *CDKN2A* hypermethylation in colorectal cancers. In fact, nearly 10% of all cases showed greater methylation in the adjacent non-neoplastic regions than in the carcinoma. Not only has age-dependent methylation been recognized as an important physiological process but recent studies show further that CpG island methylation in normal colorectal mucosa may also be related to ethnicity, tumor location and intake of supplements such as folic acid [5,8]. This indicates that methylation patterns in normal colorectal mucosa cannot be ignored supporting our decision to use normal tissue as a control in this study.

Although several different options were considered for setting a threshold value for methylation positivity, we chose to consider cases with at least 20% methylation difference between tumor and normal tissue as methylation positive. The number of positive cases, namely 20% of patients, falls within the range previously described [4]. Since background methylation in both tumor and normal tissues may reach 10%, setting a cut-off at 20% ensures that only methylated cases be assigned as positive. In addition, since we included cases enriched for >70% tumor content, it is possible that a few samples containing sufficient non-neoplastic tissue may be misclassified as methylation negative, i.e., false-negatives.

Several study groups have addressed the issue of threshold values for methylation positivity using pyrosequencing. Vasiljevic and colleagues found an optimal cut-off of 35% for methylation in prostate cancers using data resampling and statistical methods [9]. Several groups have assigned positivity to cases with a methylation density >15% [10-13]. In lymphoma, cut-offs for *CDKN2A* methylation positivity were based on receiver operating characteristics (ROC) curves and compared to the median and mean methylation levels ultimately categorized as negative, low, intermediate and high when <5%, 5-25%, 25-40% and >40% methylation was found, respectively [14]. Others have used the mean and standard deviation as a basis for cut-off value determination for CIMP-related markers [15]. These methods have advantages and drawbacks. Methods based on the mean and SD may be suboptimal since the presence of outliers, as was seen in our study, may have a considerable impact on skewing the distribution of the methylation data in normal tissues in particular. Cut-off scores derived after the analyses of entire cohorts may be disadvantageous in that they are not generalizable to other datasets. A cut-off score derived from ROC curve analysis is often advantageous as it may have the most clinically relevant value for a specific endpoint of interest, such as survival. It does nonetheless test the entire range of possible methylation values including those that may be irrelevant. Applying ROC curve analysis to our data here, we found an ‘optimal’ difference of 5% to be sufficient. This value, although statistically optimal is less compatible with the biological relevance.

In our series, *CDKN2A* methylation positivity correlated with more frequent right-sided disease, mucinous histology, tumor grade as well as with MSI, *BRAF* mutation and with *KRAS* mutation in the MSI setting only. This finding is in line with other studies showing the association of methylation with “classical” features of MSI and high-level CIMP [4]. Poorer prognosis is found in patients with CDKN2A methylation positive tumors regardless of BRAF gene status. Additionally, unfavorable survival time was again particularly observed in patients with MSS disease. These results are in line with findings from Kim et al. using pyrosequencing (n = 131), Mitomi and colleagues using q-MSP (n = 151), Liang et al. using MSP (n = 84) cases and Maeda and coworkers using q-MSP (n = 90) showing either a negative prognostic effect of *CDKN2A* methylation in univariate and/or multivariate analysis [16-19].

## Conclusion

To conclude, our study indicates that pyrosequencing for *CDKN2A* methylation analysis is robust even at low methylation levels and may be particularly suited for large solid tumor samples like colorectal cancer. However, the non-negligible methylation occurring in the adjacent normal colorectal mucosa may confound the assessment of tumor-specific *CDKN2A* hypermethylation suggesting that corresponding non-neoplastic tissue should be used as a control. This finding is certainly not only restricted to *CDKN2A* but should perhaps be considered for other genes that may be strongly methylated in normal tissues as well. The independent and highly adverse prognostic effect of *CDKN2A* methylation using the approach described here suggests that prospective analysis of this biomarker is warranted.

## Abbreviations

MSI: Microsatellite instability.

## Competing interest

The authors have no competing interests.

## Authors’ contributions

MB performed the molecular genetic studies and helped to draft the manuscript. AF carried out the laboratory experiments. AL participated in study design and coordination. IZ conceived the study, carried out the statistical analysis and manuscript drafting. All authors read and approved the final manuscript.
